# Inadequate weight gain and factors influencing it among preterm neonates in neonatal intensive care units in the Amhara region, Ethiopia, in 2022

**DOI:** 10.3389/fped.2024.1381010

**Published:** 2024-05-07

**Authors:** Yaregal Semanew, Eleny Tesfaye, Debrework Tesgera

**Affiliations:** ^1^ Paediatrics and Child Health Nursing, College of Medicine and Health Sciences, Wollo University, Dessie, Ethiopia; ^2^ School of Nursing, College of Medicine and Health Sciences, University of Gondar, Gondar, Ethiopia

**Keywords:** preterm neonates, weight gain, neonatal intensive care units, specialized hospitals, Amhara region, Ethiopia

## Abstract

**Background:**

Adequate weight gain is crucial for the health and development of preterm neonates admitted to neonatal intensive care units (NICUs). Understanding the factors influencing weight gain in this vulnerable population is essential for improving outcomes. This study aimed to assess the weight gain status and associated factors among preterm neonates admitted to NICUs in specialized hospitals in the Amhara region of Ethiopia.

**Methods:**

A cross-sectional study design involving 363 preterm neonates admitted to NICUs in specialized hospitals within the Amhara region was used. Data were collected using structured questionnaires and the Kobo Tool Box. Daily weight measurements were recorded for three consecutive days. Descriptive statistics, logistic regression analysis, and graphical presentations were utilized for data analysis and presentation.

**Results:**

The study revealed that a significant proportion (80.8%) of preterm neonates experienced poor weight gain during their NICU stay. The factors significantly associated with poor weight gain were older maternal age, delayed initiation of enteral feeding, lack of kangaroo mother care (KMC), and inadequate antenatal care visits.

**Conclusion:**

Addressing the identified factors, such as providing adequate support during the antenatal period, promoting a timely initiation of enteral feeding, and encouraging KMC practices, is crucial for improving weight gain outcomes in preterm neonates. The findings highlight the importance of a comprehensive approach to neonatal care targeting both maternal and neonatal factors. Policymakers and healthcare providers should prioritize interventions aimed at optimizing weight gain in preterm neonates to improve these neonates’ overall well-being and long-term outcomes.

## Introduction

Weight gain in the neonatal period typically begins after the first week of life, which is considered a period of physiological weight loss. With a mean period of 10.6 days, preterm neonates experience an average weight gain of 16.7 g/kg per day after reaching their birth weight (1–3). However, it is common for preterm infants, particularly those in the early stages of life, to suffer from extrauterine growth retardation, which is difficult to improve within a short period of time (4). To be discharged from neonatal intensive care units (NICUs), preterm neonates are expected to gain an average of 15–20 g/kg of their body weight after the physiological weight loss period. The sustained weight gain within this range for three consecutive days is one of the criteria for determining if a preterm neonate is ready for discharge (5–8).

Early postnatal growth failure is highly prevalent among preterm and very low birth weight (LBW) neonates and is influenced by a complex array of factors that affect their overall growth and development (1). Low- and middle-income countries often face a significant burden of poor fetal and neonatal growth, which may be attributed to inadequate care both in NICUs and at home (2, 9). Beyond the neonatal period, there is a substantial, but unquantified burden of small-for-gestational-age infants who are at an increased risk of experiencing long-term morbidity in childhood, such as poor linear growth, and chronic non-communicable diseases in adulthood (1, 9).

Despite numerous interventions aimed at optimizing preterm infant nutrition, achieving adequate weight gain has remained challenging and has resulted in preterm infants often being underweight at the time of hospital discharge (10, 11). However, interventions implemented in NICUs have shown promise in facilitating a faster achievement of full feeding, increasing neonatal weight gain, and shortening the length of hospital stay for admitted preterm babies (12). The optimal growth pattern for preterm neonates, taking into consideration their future neurological, cardiovascular, and metabolic development outcomes, remains largely unknown and warrants further investigation (3, 13).

While it is normal for infants to experience weight loss during the first week of life, poor weight gain beyond this period poses a significant problem for preterm neonates receiving care in NICUs (14). In the context of NICUs in Ethiopia, the postnatal weight gain of preterm neonates may be influenced by various factors, including preterm feedings in the NICU, timing of initiation of full feeding, and presence of comorbidities. This study aims to investigate the weight gain status and the factors influencing it in preterm neonates receiving care in the NICUs in our locality.

## Methods

### Study design, setting, and period

An institution-based cross-sectional study was performed on preterm neonates admitted in NICUs paired with their mothers in comprehensive specialized hospitals (CSHs) in the Amhara region, Ethiopia, from 1 October to 10 November 2022 (Ref. No. 034/2015).

The study was conducted on preterm neonates who were admitted to the NICUs of CSHs in the Amhara region, Ethiopia. This work involved seven hospitals, except for the Woldia Hospital for the pilot study. Hospitals having NICUs are organized with neonatal beds, necessary materials, equipment, health professionals, and other staff members. The major services offered by these hospitals are general neonatal care, blood and exchange transfusion, phototherapy, kangaroo mother care (KMC), and ventilation support, such as continuous positive airway pressure (15).

The participating hospitals were as follows: University of Gondar Comprehensive Specialized Hospital (UoGCSH), which has an annual admission of 726 preterm and LBW neonates; Debretabor Comprehensive Specialized Hospital (DTCSH), which has 512 preterm and LBW neonates yearly; Dessie Comprehensive Specialized Hospital (DCSH), which has more than 200 neonates per month; Debre Birhan Comprehensive Specialized Hospital (DBCSH); Debremarkos Comprehensive Specialized Hospital (DMCSH), which has 612 preterm and LBW neonates yearly; Tibebe Ghion Comprehensive Specialized Hospital (TGCSH), which has 690 preterm and LBW neonates; and Felegehiwot Comprehensive Specialized Hospital (FHCSH), which has 720 preterm and LBW neonates. According to estimated reports, the NICU of each hospital had an average admission of 75–100 preterm neonates per month (15–17).

### Population

The source population was all preterm neonates admitted to the NICUs of the CSHs in the Amhara region. The study population was all preterm neonates paired with their mothers who were available during the data collection period.

### Inclusion and exclusion criteria

All preterm neonates admitted for more than 24 h to the NICUs of the CSHs in the Amhara region were included in the study.

Preterm neonates with a chronological age (age after birth) of less than 7 days and those with confirmed major congenital anomalies and visible generalized edema were excluded.

### Sample size determination and sampling procedure

The sample size was determined by the following single population proportion formula after conducting a pilot study at Woldia Comprehensive Specialized Hospital and by considering the proportion of preterm neonates with poor weight gain (*p* = 68.75%), 95% confidence level, and 5% margin of error: *n* = Z*α*/2^2^ × *p* × (1−*p*)/*d*^2^, where Z*α*/2 is the critical value of the normal distribution at *α*/2% and 95% confidence levels; *p* is the proportion of poor weight gain; and *d* is the margin of error [*n* = (1.96)^2^ × 0.687(1–0.687)/(0.05)^2^ = 330.4].

The final sample size, including the 10% non-response rate, was 363.

Based on the calculated sample size, the study samples for each hospital were proportionally allocated, and systematic random sampling was used in each NICU. The samples were picked by calculating the *K*-value from the actual number of admissions to each hospital. Based on the monthly hospital reports and the proportions for each, the approximate *K* interval was two.

**Figure 1:**
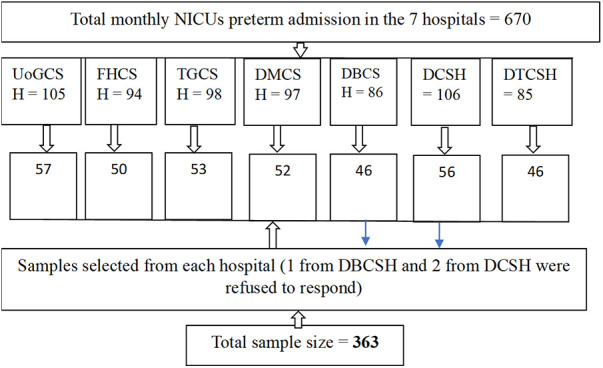
Schematic presentation of sampling in the NICUs of the CSHs at the Amhara region, Ethiopia, in 2022.

The recent estimated total monthly NICU admissions of the hospitals were as follows: 270 for UoGCSH; 198 for FHCSH; 200 for TGCSH; 196 for DTCSH; 250 for DMCSH; 265 for DCSH; and 190 for DBCSH. From these reports, the number of preterm admissions was identified from each hospital, and the samples were proportionally allocated (Figure 1).

### Variables of the study

The dependent variable was the weight gain status of preterm neonates (adequate weight gain or poor weight gain).

The independent variables were maternal sociodemographic and obstetric characteristics (i.e., age, educational status, occupation, residence, antenatal care (ANC) visits, history of preterm birth, parity, delivery place, and mode of delivery), neonatal characteristics and care in the NICUs (i.e., age, sex, birth weight, gestational age, and KMC), feedings and comorbidity in the NICUs (i.e., initiation to first feed, feeding type, method of feeding, feeding frequency, age of reaching full feeding, and comorbid conditions), and maternal knowledge on preterm feeding.

### Operational definitions

#### Poor weight gain

A preterm neonate aged ≥7 days with an average daily weight gain of <15 g/kg/day (calculated from the average weight gain of three consecutive days) (8, 18).

#### Adequate weight gain

A preterm neonate aged ≥7 days with an average daily weight gain of ≥15 g/kg/day (calculated from the average weight gain of three consecutive days) (18, 19).

#### Age of reaching full feeding

The age in days a neonate starts to get the total daily required feed, including oral milk and IV fluids (140–150 ml/kg/day volume or 110–120 kcal/kg/day if written in calories) (8).

#### Preterm baby

A baby born at less than 37 completed weeks of gestation and determined by the physician at admission using first-trimester ultrasound and/or last normal menstrual period (picked from the chart).

#### Comorbid conditions

Common medical diagnosis of preterm neonates in NICUs [respiratory distress syndrome (RDS), neonatal sepsis, necrotizing enterocolitis (NEC), jaundice, and hypothermia] (19).

#### Maternal knowledge on preterm feeding

Measured by asking six questions about preterm feeding and breastfeeding. A mother is said to have good knowledge if she answers four or more questions; otherwise, she is considered to have poor knowledge (14).

#### Major congenital anomalies

Major congenital abnormalities that may cause weight change due to edema or feeding difficulties (i.e., hydrocephalus, meningoceles, and congenital heart disease identified and diagnosed by physicians) (19).

### Data collection tools and procedures

Data were collected by using a data review sheet for the neonatal characteristics obtained from charts and daily care records in NICUs. The initial weight of each selected neonate was picked from daily records (most hospitals in the study area record the daily morning weight of preterm neonates with a digital weighing scale). The neonates were weighed if not already recorded. The weight record was then registered for three consecutive days with a digital weighing scale specified as Hopkins (20) at 2:00–3:00 a.m. local time after a diaper change. A paper and/or glove case was placed on the weighing tray, and a button was pressed to adjust the reading to 0 and reduce the measurement bias before the baby was weighed. A semi-structured questionnaire derived from related literature and World Health Organization recommendations in NICUs, which included six questions to assess the mothers' knowledge on preterm feeding and translated from English to Amharic, was used to interview the mothers (14). One BSc neonatal nurse, who had work experience and could speak the local language of each hospital, collected the data. An ethical approval letter was obtained from the IRB of the University of Gondar (ref. no. 034/2015), and permission letters were obtained from each hospital. Additionally, verbally informed consent was taken from the mothers for the interviews and for use in reviewing the neonates' charts.

To ensure the data quality, a pretest at Borumeda Hospital was conducted on 18 samples before the pilot study and the actual data collection period. The data collectors and supervisors were given 1-day training about the data collection tool and how to record the weight of neonates. Daily supervision was performed by the principal investigator and assigned supervisors. Data quality was checked for consistency.

### Data processing and analysis

Data were exported to an Excel sheet from the Kobo Tool Box data collection software. These were then cleaned, checked, coded, and exported to Stata version 16 for further analysis. Descriptive statistics were presented by frequencies, tables, and figures. The weight gain status of the preterm neonates was computed as poor weight gain and adequate weight gain based on the minimum daily weight gain of the preterm neonates from the national NICU guideline (19). Binary logistics regression was fitted. Model fitness was checked through the Hosmer–Lemeshow test (prob > *χ*^2^ = 0.6375). Multicollinearity was also checked (VIF = 1.41). Bivariable and multivariable analyses were performed to determine the variables that were significantly associated with the weight gain status of the preterm neonates in the NICUs. The level of statistical significance was declared at a *p*-value < 0.05 with an adjusted odds ratio ≠ 1 at a 95% confidence interval (CI).

## Results

### Sociodemographic and obstetric characteristics of mother-related factors

The participants involved in this study were 360 preterm neonates paired with their mothers. The response rate was 99%. The mean ± standard deviation (SD) of the age of mothers was 27.43 ± 5.6 years. Almost more than half (59.44%) of the mothers were urban residents, and 28.8% were primipara, that is, it was their first time giving birth (Table 1).

**Table 1:** Maternal sociodemographic and obstetric characteristics in the NICUs of the CSHs in the Amhara region, Ethiopia, in 2022.

Variables	Categories	Frequency	Percentage
Mothers’ age			
	15–24	123	34.17
	25–29	98	27.22
	30–49	139	38.61
Residence			
	Rural	146	40.56
	Urban	214	59.44
Mothers’ educational level			
	No formal education	120	33.33
	Elementary education	18	5.00
	Secondary education	190	52.78
	College and above	32	8.89
Mothers’ occupation			
	Government employed	74	20.56
	Housewife	208	57.78
	Private employee	28	7.78
	Self-employee	50	13.89
Parity			
	Primipara	104	28.89
	Multiparous	256	71.11
Previous history of preterm birth			
	No	329	91.39
	Yes	31	8.61
Place of delivery			
	Health facility	323	89.72
	Home	37	10.28
Mode of delivery			
	Cesarean section	67	18.61
	Instrumental delivery	4	1.11
	Spontaneous vaginal delivery	289	80.28

### Neonatal characteristics and care-related factors

**Figure 2:**
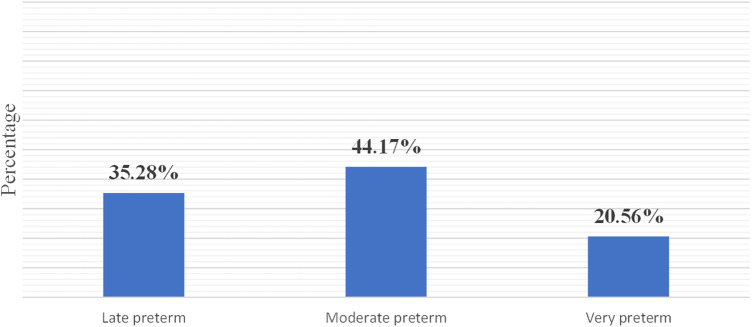
Gestational age category of the preterm neonates in the NICUs of the CSHs in the Amhara region, Ethiopia, in 2022.

The mean ± SD of the age of the preterm neonates included in this study was 12.39 ± 5 days. Out of 360 neonates, 20.56% were very preterm (i.e., born before 32 weeks of gestation), and 47.22% were on KMC (Table 2 and Figure 2).

**Table 2:** Characteristics of and care for the preterm neonates in the NICUs of the CSHs in the Amhara region, Ethiopia, in 2022.

Variables	Categories	Freq.	Percentage
Sex of the neonate			
	Female	175	48.61
	Male	185	51.39
Birth weight			
	Very low birth weigh	109	30.28
	Low birth weight	251	69.72
Kangaroo mother care			
	No	190	52.78
	Yes	170	47.22

### Feeding characteristics and comorbidity-related factors

Approximately half (53.89%) of the preterm neonates in the NICUs were fed via a nasogastric tube; 44.72% started enteral feeding within 24 h of birth; 66.94% started getting full feeding within 7 days of age; and 28% were diagnosed with RDS (Table 3).

**Table 3:** Feeding characteristics and comorbidities of the preterm neonates in the NICUs of the CSHs in the Amhara region, Ethiopia, in 2022.

Variables	Categories	Freq.	Percentage
Start of first feeding			
	After 24 h	199	55.28
	Within 24 h	161	44.72
Method of feeding			
	Cup feeding	37	10.28
	Directly breastfeeding	63	17.50
	NG tube feeding	194	53.89
	Parenteral feeding (IV fluids only)	66	18.33
Frequency of feeding			
	Feeding as needed (on breast)	32	8.89
	Every 2 h	103	28.61
	Every 3 h	225	62.50
Age of starting full feeding	Within 7 days	241	66.94
	After 7 days	119	33.06
Comorbidities			
Respiratory distress syndrome			
	No	259	71.94
	Yes	101	28.06
Neonatal sepsis			
	No	165	45.83
	Yes	195	54.17
Necrotizing enterocolitis			
	No	315	87.50
	Yes	45	12.50
Jaundice			
	No	254	70.56
	Yes	106	29.44
Hypothermia			
	No	215	59.72
	Yes	145	40.28

### Weight gain status of preterm neonates in the NICUs

**Figure 3:**
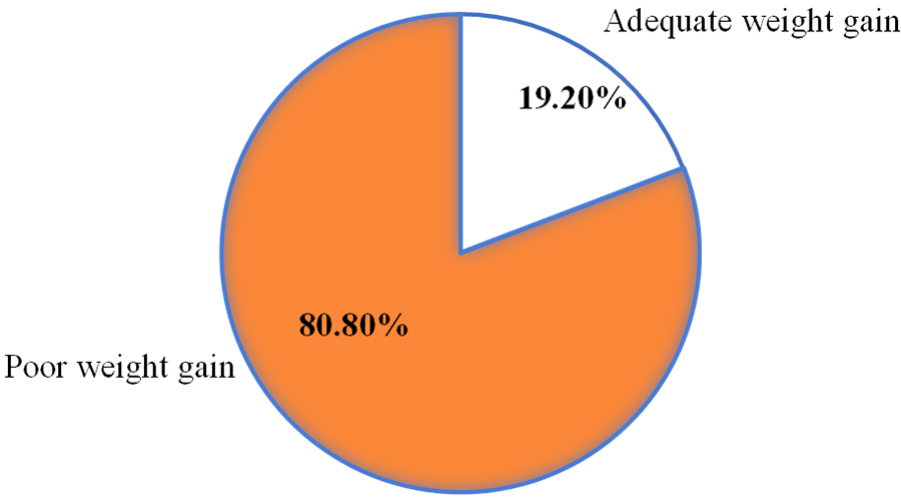
Weight gain status among the preterm neonates in the NICUs of the CSHs in the Amhara region, Ethiopia, in 2022.

The average daily weight gain of the preterm neonates included in this study was 10.16 ± 5.32 g/kg of body weight. The overall proportion of the preterm neonates who gained an average daily weight below the minimum daily weight gain of the national NICU guideline was 80.8% (95% CI, 76.74, 84.91) (Figure 3).

### Factors associated with weight gain status of preterm neonates in the NICUs

Thirteen variables, namely, maternal age, residence, ANC visits, knowledge on preterm feeding, sex of the neonate, age of the neonate, birth weight, start time of feeding, feeding frequency, age of starting full feeding, KMC, jaundice, and RDS had a *p*-value < 0.2 in the bivariable logistics regression analysis. In the multivariable logistics regression analysis, the variables of maternal age, ANC visit, time of starting enteral feeding, and KMC were significantly associated with the weight gain status of the preterm neonates in the NICUs.

Mothers of older age (30–49 years) were 2.8 times more likely to have preterm neonates with poor weight gain than those aged 25–29 years [AOR = 2.83 (95% CI, 1.28, 6.25)]. Mothers who had no ANC visit and only one ANC visit were almost six times more likely to have preterm neonates with poor weight gain than those who had three or more ANC visits [AOR = 5.69 (95% CI, 1.24, 26.01) and AOR = 5.8 (95% CI, 1.30, 25.54), respectively]. The preterm neonates who started their enteral feeding after 24 h were 3.8 times more likely to have poor weight gain than those who started their enteral feeding within 24 h [AOR = 3.76 (95% CI, 1.50, 9.42)]. The preterm neonates who did not receive KMC were three times more likely to have poor weight gain than those who received the care [AOR = 3.08 (95% CI, 1.28, 7.43)] (Table 4).

**Table 4:** Factors associated with the weight gain status among the preterm neonates in the NICUs of the CSHs in the Amhara region, Ethiopia, in 2022.

Variables	Category	Poor weight gain		COR 95% CI	AOR 95% CI	*p*-value
		No (*n*)	Yes (*n*)			
Mothers’ age	15–24	24	99	1.65 (0.88, 3.08)	1.00 (0.45, 2.21)	0.99
	25–29	28	70	1	1	** **
	30–49	17	122	2.87 (1.46, 5.61)	2.83 (1.28, 6.25)	**0**.**01**
Residence	Urban	60	154	1	1	
	Rural	9	137	5.93 (2.83, 12.39)	2.58 (0.95, 6.96)	0.06
ANC visits	No visit at all	6	87	3.29 (0.92, 11.80)	5.69 (1.24, 26.01)	**0**.**02**
	1 visit	20	119	1.35 (0.45, 3.98)	5.80 (1.30, 25.54)	**0**.**02**
	2 visits	20	63	0.37 (0.13, 1.07)	3.41 (0.72, 16.02)	0.11
	3 and more visits	5	22	1	1	
Mothers’ knowledge on preterm feeding	Good knowledge	53	143	1	1	
	Poor knowledge	16	148	3.42 (1.87, 6.27)	1.84 (0.87, 3.89)	0.10
Neonates’ age	–	–	–	1.03 (0.98, 1.09)	0.98 (0.91, 1.05)	0.60
Sex of the neonate	Female	29	146	1.38 (0.81, 2.36)	1.51 (0.79, 2.86)	0.20
	Male	40	145	1	1	
Birth weight	Very low birth weight	15	94	1.71 (0.92, 3.20)	0.46 (0.17, 1.20)	0.11
	Low birth weight	54	197	1	1	
Started enteral feeding	After 24 h	13	186	7.63 (3.9, 14.60)	3.76 (1.50, 9.42)	**0**.**005**
	Within 24 h	56	105	1	1	
Frequency of feeding	Breastfed as needed	8	24	1	1	
	Every 2 h	34	69	0.67 (0.27, 1.66)	0.70 (0.23, 2.10)	0.53
	Every 3 h	27	198	2.44 (0.99, 5.98)	1.36 (0.43, 4.30)	0.59
Started full feeding	Within 7 days	58	183	1	1	
	After 7 days	11	108	3.11 (1.56, 6.18)	0.92 (0.34, 2.49)	0.88
KMC	No	13	177	6.68 (3.49, 12.78)	3.08 (1.28, 7.43)	**0**.**01**
	Yes	56	114	1	1	
Jaundice	No	41	213	1	1	
	Yes	28	78	0.53 (0.31, 0.92)	1.15 (0.56, 2.34)	0.70
RDS	No	56	203	1	1	
	Yes	13	88	1.86 (0.97, 3.58)	1.11 (0.43, 2.85)	0.81

KMC, kangaroo mother care; RDS, respiratory distress syndrome.

Numbers in bold are to highlight statistical significance.

## Discussion

This study aimed to assess the weight gain status of preterm neonates admitted to the NICUs in CSHs in the Amhara region, Ethiopia. The proportion of the preterm neonates with poor weight gain was 80.8% (95% CI, 76.74, 84.91). This finding was slightly lower than that in a study conducted in Tanzania (86.8%) (14) and higher than that conducted in Uganda, wherein 48.1% of neonates did not regain their birth weight by day 21 (5). The possible reason for this result might be the current study's exclusion of the preterm neonates whose age was less than 7 days and the Tanzanian study's inclusion of those neonatal age groups. The first week of the neonatal period is a time of physiological weight loss of up to 15% of their birth weight for preterm neonates (8). Even though the value obtained in the current study is slightly lower in figure, it is still high compared to that in the Tanzanian study because it did not include neonates who were in the physiological weight loss period. The reason behind this might be the differences in the neonatal intensive care unit levels, available materials in the unit, or quality of care differences. A possible reason for the difference with that of the study in Uganda might be their usage of a weight record of 21 days and a slight difference in the study objectives.

However, in the current finding, the proportion of the preterm neonates with an average daily weight gain below the minimum daily weight gain requirement of the national neonatal intensive care unit guideline is very high and can be a significant problem in the NICUs.

Regarding factors determining the weight gain status of the preterm neonates, the current study showed that the delayed start of enteral feeding was significantly associated with poor weight gain. Preterm neonates who started enteral feeding after 24 h were almost four times more likely to have poor weight gain compared to those who started enteral feeding within 24 h. This result was supported by studies conducted in the US (21), Romania (22), India (23), Tanzania (14), and Uganda (5) and might be caused by the delayed start of enteral feeding possibly influencing the achievement of the full daily requirement and affecting the weight gain status.

On the contrary, another global study showed that the delayed start of enteral feeding only yields a minimal difference in the weight gain status of preterm neonates and that the introduction of enteral feeds is usually completed after 4–10 days for neonates with a GA of 32–34 and <27 weeks while they are on parenteral feeding (3). The possible reason for this discrepancy might be the study's indication that neonates were on parenteral nutrition, which may include intravenous proteins, lipids, or other colloid products that are not currently available for our setup in the study area. Another difference might be that the study was conducted in five international NICUs and neonatal care centers that included healthy neonates.

In this work, KMC was another factor significantly associated with the weight gain status of the preterm neonates in the NICUs. The preterm neonates who did not receive KMC were three times more likely to have poor weight gain compared to those who received it. This finding is supported by a study conducted in Egypt (24) and one other systematic review (25). Similarly, a meta-analysis of 11 articles conducted in Indonesia showed that KMC increased the weight gain in premature infants compared to those who were under a conventional method of care (26). This might be due to the fact that KMC prevents neonatal heat loss and may reduce the neonates' calorie expenditure to maintain their body temperature, which leads to a maintained body weight (8). Additionally, KMC establishes breastfeeding and enhances frequent feeding as the baby stays with the mother for a longer period.

Another systematic review indicated that further research is needed to obtain strong evidence on the significant association between the weight gain status of sick preterm neonates in NICUs and KMC (27). This might be due to the lack of a universal consensus as regards whether or not sick and unstable preterm neonates like those on oxygen and other interventions must be placed on KMC.

Another significantly associated variable in this study was the ANC visit of the mother during her current pregnancy, in which neonates born to mothers who had less than two ANC visits were almost six times more likely to have poor weight gain compared to those born to mothers who had three or more ANC visits. Even though the investigator failed to find directly related studies supporting or contradicting the current finding, there were studies indicating that the overall health outcome of neonates born to mothers who had at least four ANC visits is better than those born to mothers who had no visit or only few ANC visits. The neonatal morbidity and mortality were also higher among neonates born to mothers who had no visit or fewer ANC visits compared to their counterparts (28–30).

Preterm neonates born to mothers whose ages range from 30 to 49 years were three times more likely to have poor weight gain compared to those born to mothers aged 25–29 years. This finding lacks related supporting or contradicting evidence from other studies, but some studies have shown that neonatal outcomes among neonates born to mothers in both extreme ages (i.e., maternal age <16 years and maternal age >35 years) were poor compared to those born to mothers in the medium age group (i.e., maternal ages: 20–35 years) (31, 32).

The delayed time of starting enteral feeding was a clinically associated variable in relation to the fact that a delay in starting feeding may delay the realization of early full feeding in NICUs. Consequently, this may lead to poor weight gain in preterm neonates. Therefore, early commencement and monitoring of enteral feeding in the NICUs may help in maintaining adequate weight gain of preterm neonates.

## Conclusion and recommendations

The proportion of poor weight gain among preterm neonates admitted to the NICUs of CSHs in the Amhara region was very high. Older maternal age, less than two ANC visits of the mother, delayed starting of enteral feeding, and not getting KMC were significantly associated variables.

The findings of this study have important implications for neonatal care in resource-limited settings and can contribute to the development of targeted interventions to improve weight gain among preterm neonates.

Health care providers working in ANC clinics and other maternal care centers should provide adequate ANC counseling to increase the uptake of ANC visits, including counseling on newborn care contents, and risks of early and late pregnancies related to maternal age. Health professionals working in NICUs should facilitate KMC practices for preterm neonates and monitor early enteral feedings.

## Data Availability

The raw data supporting the conclusions of this article will be made available by the authors, without undue reservation.
